# Supplementary material for: Glycolysis regulates KRAS plasma membrane localization and function through defined glycosphingolipids
Source: Nat Commun. 2023 Jan 28;14:465. doi: 10.1038/s41467-023-36128-5 (PMC9884228; doi:10.1038/s41467-023-36128-5)
Supplement: Supplementary file 1 — Supplementary Information [file 41467_2023_36128_MOESM1_ESM.pdf]

Figure S1

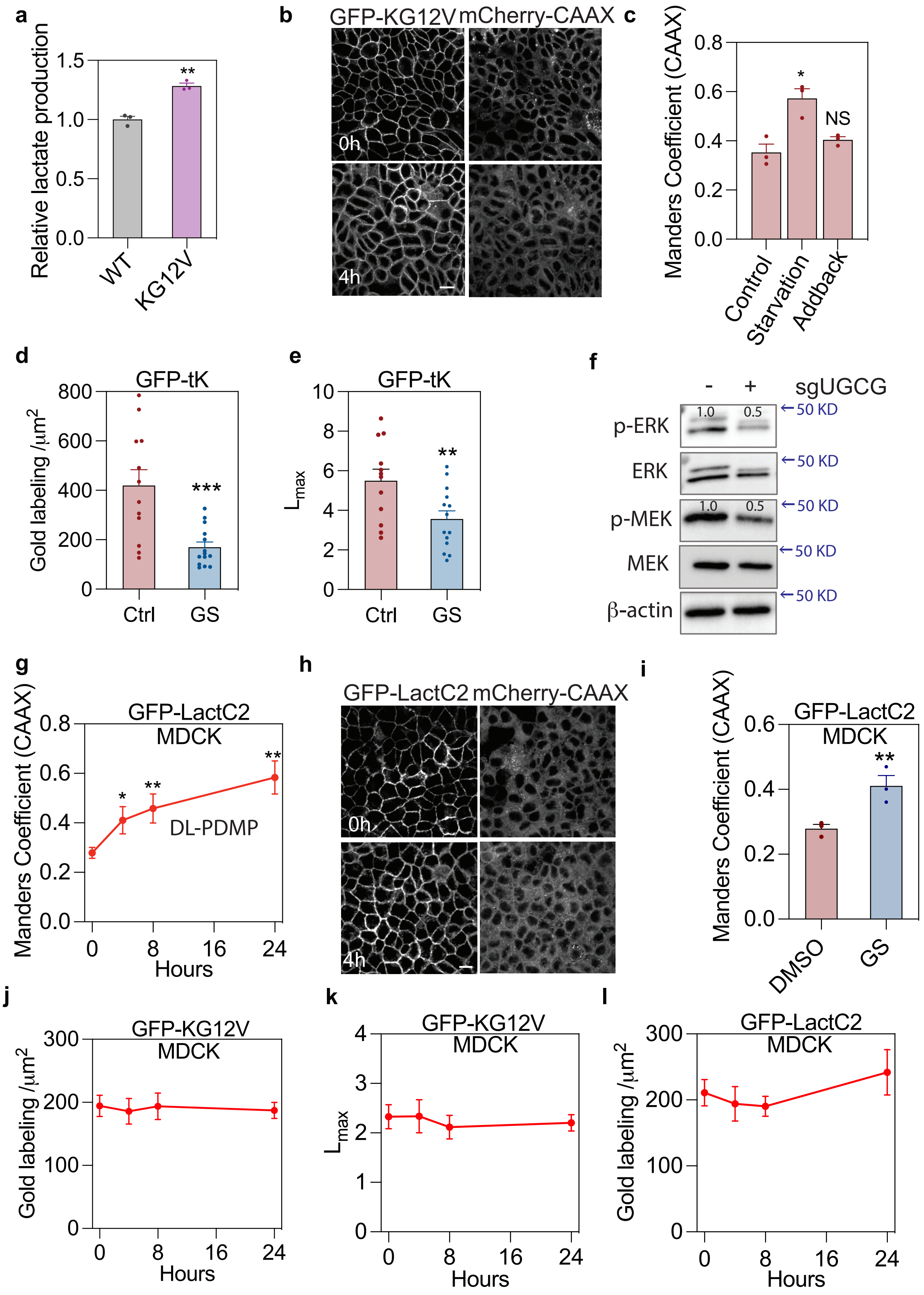

a
Relative lactate production
1.5
1.0
0.5
0.0
**
WT
KG12V
b
GFP-KG12V
mCherry-CAAX
0h
4h
c
Manders Coefficient (CAAX)
0.8
0.6
0.4
0.2
0.0
*
NS
Control
Starvation
Addback
d
GFP-tK
Gold labeling /μm2
800
600
400
200
0
***
Ctrl
GS
e
GFP-tK
Lmax
10
8
6
4
2
0
**
Ctrl
GS
f
-
+
sgUGCG
p-ERK
1.0
0.5
←50 KD
ERK
←50 KD
p-MEK
1.0
0.5
←50 KD
MEK
←50 KD
β-actin
←50 KD
g
GFP-LactC2
MDCK
Manders Coefficient (CAAX)
0.8
0.6
0.4
0.2
0.0
*
**
**
DL-PDMP
0
8
16
24
Hours
h
GFP-LactC2
mCherry-CAAX
0h
4h
i
GFP-LactC2
MDCK
Manders Coefficient (CAAX)
0.6
0.4
0.2
0.0
**
DMSO
GS
j
GFP-KG12V
MDCK
Gold labeling /μm2
300
200
100
0
0
8
16
24
Hours
k
GFP-KG12V
MDCK
Lmax
4
3
2
1
0
0
8
16
24
Hours
l
GFP-LactC2
MDCK
Gold labeling /μm2
300
200
100
0
0
8
16
24
Hours

Figure S1. Effects of glucose starvation are mediated by the minimal KRAS anchor.

(**a**) Relative lactate production rate of WT and KRASG12V expressing MDCK cells. Data are mean ± SD; n=3 wells; two-tailed Student's t-tests, $^{**}p<0.01$. (**b**) Representative confocal images of MDCK stably expressing GFP-KRASG12V and mCherry-CAAX (an endomembrane marker) cultured in glucose-depleted medium for 4hr. Scale bar: 20 μm. (**c**) MDCK cells stably expressing GFP-KRASG12V and mCherry-CAAX were incubated in regular, or glucose depleted medium for 4h, then returned to glucose replete medium for 1h. Colocalization of GFP-KRASG12V and mCherry-CAAX was analyzed using Manders coefficients (± SD, n =4 confocal images). Statistical significance of differences from control cells were evaluated in t-tests ($^{*}p < 0.05$, NS: non-significant). (**d**, **e**) MDCK cells stably expressing GFP-tK were maintained in regular or glucose-depleted medium for 4h. Intact PM sheets prepared from the cells were fixed, gold labeled, imaged, and analyzed as in Figure 1. GFP-tK plasma membrane (PM) binding was quantified as mean gold labeling density (mean ± SEM) (**d**). Nanoclustering was quantified as the maximum value of the *L(r)-r* function (=*Lmax*) (**e**). (**f**) Immunoblot analysis for the total or phosphorylated abundance of indicated proteins. Cell lysates were prepared from KRASG12V-expressing Caco-2 cells depleted of UGCG (sgUGCG) or expressing control vectors (sgCtrl). Intensities of p-ERK and p-MEK were analyzed by ImageJ and normalized to total-ERK and MEK respectively. The experiment was repeated 3 times. Uncropped gels of replicates are provided in a source data file. (**g**) PM levels of GFP-LactC2 in MDCK cells treated with DL-PDMP for 4h. Data are mean ± SD; n = 3; two-tailed Student's t-tests, $^{*}p<0.05$, $^{**}p<0.01$. (**h**) Representative confocal images of MDCK stably expressing GFP-LactC2 and mCherry-CAAX cultured in glucose-depleted medium for 4h. Scale bar: 20 μm. (**i**) PM mislocalization of GFP-LactC2 evaluated using Manders coefficients (±SD, n≥4 independent relicates, two-tailed Student's t-test, $^{*}p<0.05$). (**j**-**l**) PM localization or nanoclustering of MDCK cells expressing KRASG12V (n = 16) or LactC2 (n = 15) and treated with DMSO over a period of 24h (mean ± SEM). For (**e**, **k**), nanoclustering was

quantified as the maximum value (*Lmax*, ± SEM) of the *L(r)-r* function. Significance differences from control cells at each time point were evaluated using bootstrap tests (**$p < 0.01$). Differences in gold labeling densities (**d**, **j**, **l**) were evaluated using two tailed Student's t-tests (***$p < 0.001$). Exact p-values and source data are provided as a Source Data file.

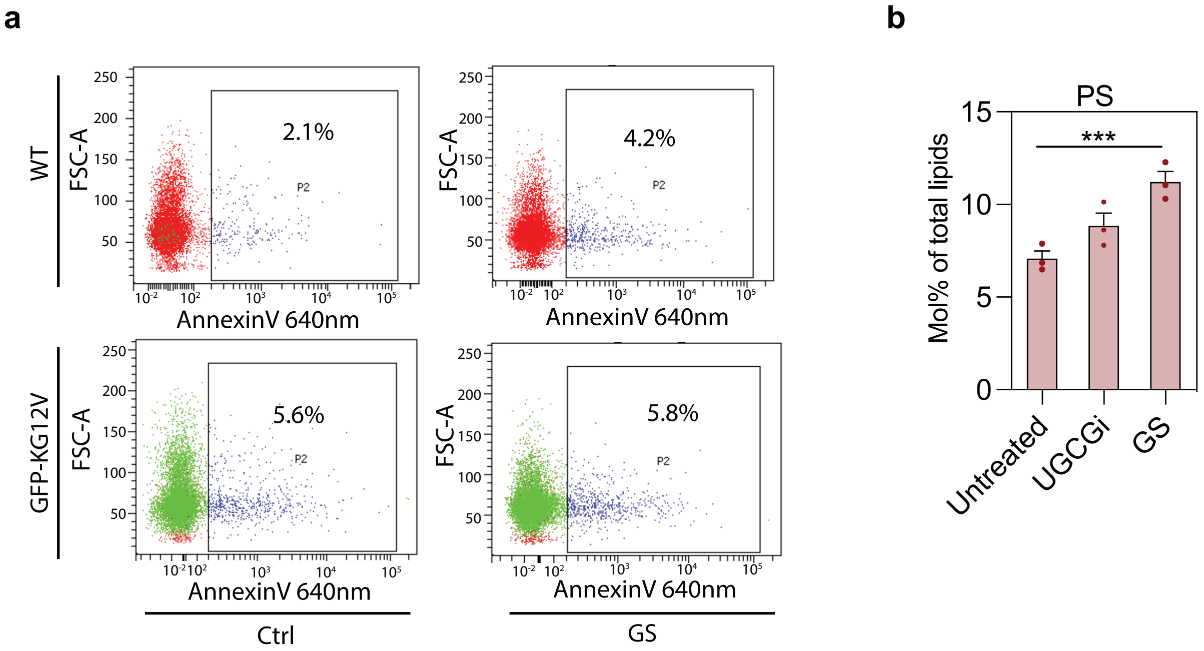

Figure S2. PtdSer lost from the inner PM upon glucose starvation is not flipped to the outer PM leaflet

(a) WT or GFP-KRASG12V-expressing MDCK cells were treated with control, or glucose-depleted (GS) media, for 4h before staining with fluorescently-labeled annexin V and counting by flow-cytometry. The percentage of cells displaying PtdSer on the outer PM leaflet, as evidenced by annexin V staining, is shown for each condition. (b) Lipidomic analysis of PtdSer (mol%) in KRASG12V-expressing MDCK cells incubated in regular or glucose depleted medium for 4hr, or treated with 25mM DL-PDMP (UGCGi) for 24 hr. Data are mean ± SD; n=3 independent replicates; differences were evaluated in two-tailed Student's t-tests, ***$p<0.001$.

Figure S3

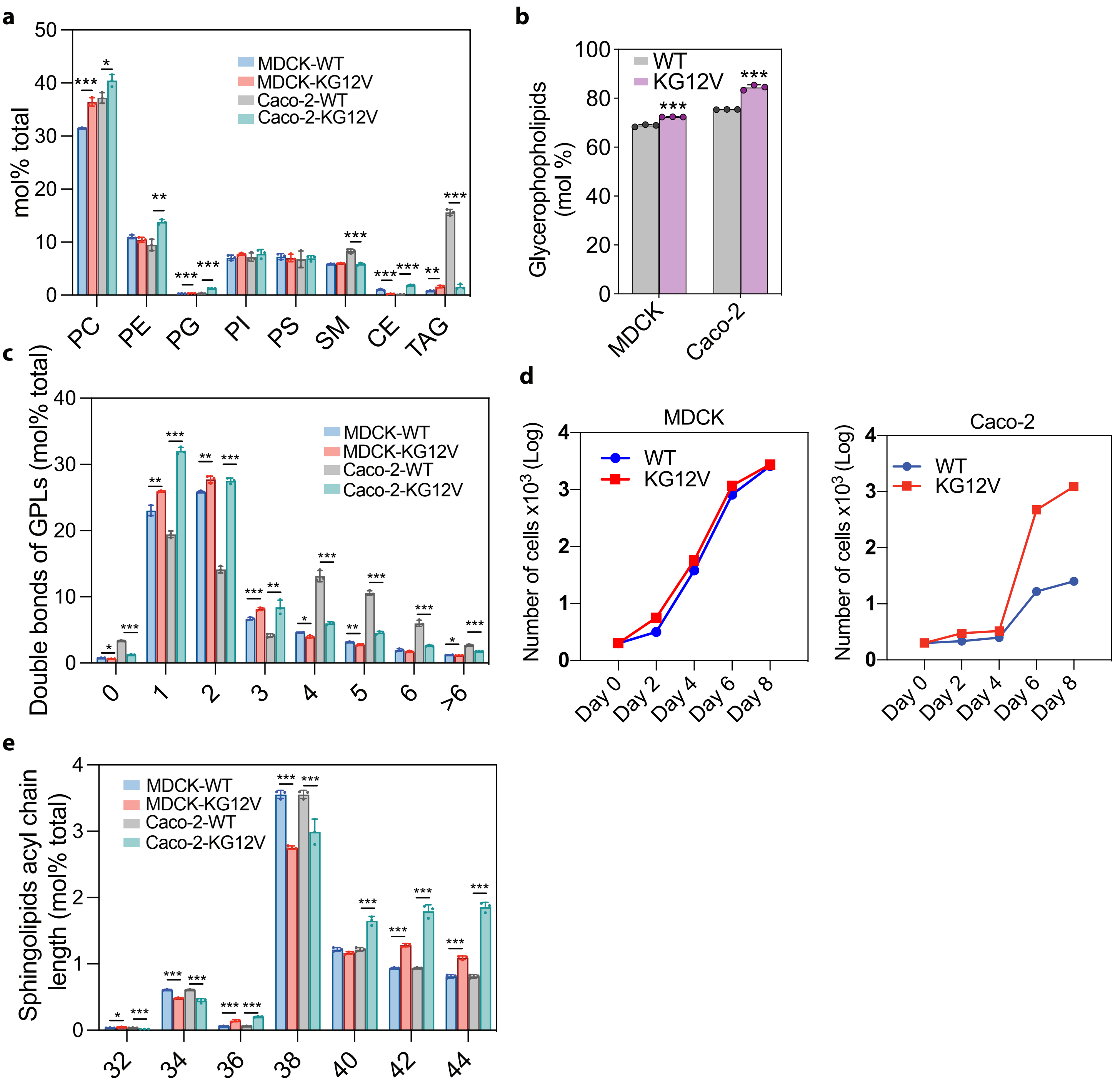
a
mol% total
MDCK-WT
MDCK-KG12V
Caco-2-WT
Caco-2-KG12V
PC
PE
PG
PI
PS
SM
CE
TAG
b
Glycerophopholipids (mol %)
WT
KG12V
MDCK
Caco-2
c
Double bonds of GPLs (mol% total)
MDCK-WT
MDCK-KG12V
Caco-2-WT
Caco-2-KG12V
0
1
2
3
4
5
6
>6
d
MDCK
Caco-2
Number of cells x10^3 (Log)
WT
KG12V
Day 0
Day 2
Day 4
Day 6
Day 8
e
Sphingolipids acyl chain length (mol% total)
MDCK-WT
MDCK-KG12V
Caco-2-WT
Caco-2-KG12V
32
34
36
38
40
42
44

Figure S3. KRASG12V expression alters acyl chain structures of different lipid species

(**a**-**c** and **e**) lipidomic analyses using shotgun mass spectrometry (MS) of wild type (WT), or GFP-KRASG12V-expressing (KG12V) MDCK, or Caco-2 cells: (**a**) comparison of lipid classes, (**b**) total molar percentages (Mol%) of glycerophospholipids (mean ± SD), (**c**) Mol% of glycerophospholipids with different degrees of acyl chain desaturation, shown as number of double bonds. (**d**) Growth curves of WT and KRASG12V-expressing MDCK and Caco-2 cells. (**e**) Comparison of total sphingolipids with different acyl chain length (= total length of both acyl chains combined). Differences between the KRASG12V-expressing and cognate WT cell lines in panels **a**-**e** were evaluated in t-tests (*$p < 0.05$).

Figure S4

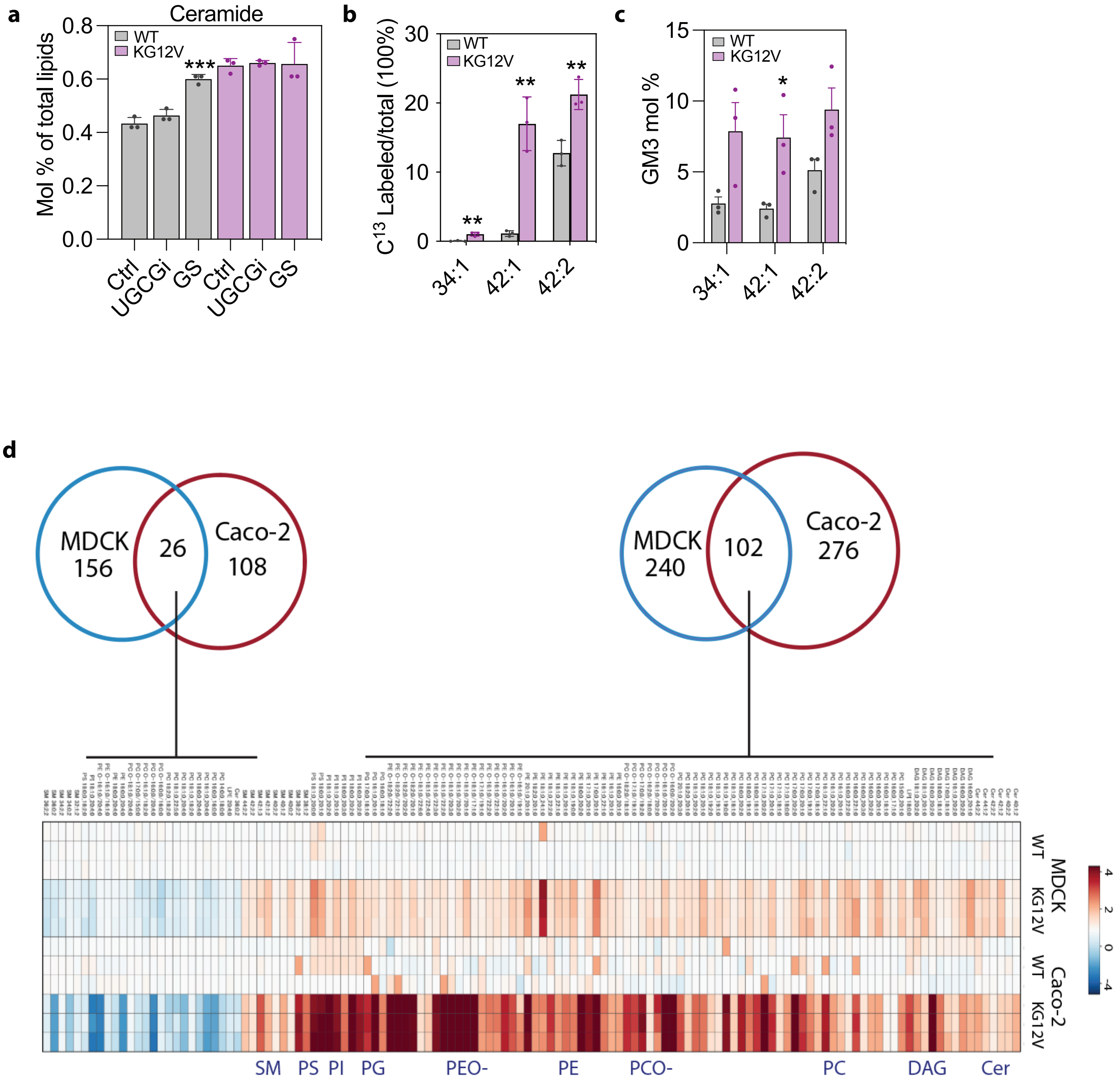
a
Ceramide
Mol % of total lipids
0.8
0.6
0.4
0.2
0.0
WT
KG12V
***
Ctrl
UGCGi
GS
Ctrl
UGCGi
GS
b
C^{13} Labeled/total (100%)
30
20
10
0
WT
KG12V
**
**
**
34:1
42:1
42:2
c
GM3 mol %
15
10
5
0
WT
KG12V
*
34:1
42:1
42:2
d
MDCK
156
26
Caco-2
108
MDCK
240
102
Caco-2
276
MDCK
WT
KG12V
Caco-2
WT
KG12V
4
2
0
-2
-4
SM
PS
PI
PG
PEO-
PE
PCO-
PC
DAG
Cer

Figure S4. KRASG12V expression reprograms the cellular lipidome

(**a**) Ceramide levels shown as mean mol% in glucose depleted (GS), or DL-PDMP treated WT and KRASG12V expressing MDCK cells (mean ± SD; n = 3 independent replicates). (**b**) Relative abundance of HexCer species, shown as mol% (mean ± SD, n=3 independent biological replicates), with different acyl chain length (= total length of both acyl chains combined, and number of double bonds) that were newly synthesized from [U-$^{13}C_6$]-glucose after 4h incubation in WT or KRASG12V- expressing MDCK cells. (**c**) Further analysis of data in Fig. 3F showing relative abundance (mean mol% ± SD, n=3 independent biological replicates) of the major species of GM3 by acyl chain structure in WT or KRASG12V- expressing MDCK cells. For **a-c**, two-tailed Student's t-tests were used to evaluate the differences between respective lipid species (*p<0.05, **p<0.01, ***p<0.001). (**d**) Heat map of shared changes in lipid species of MDCK and Caco-2 cells upon KRASG12V expression with red and blue colors indicating an increase and decrease, respectively. Data are shown as $\log_2$ fold changes for n=3 independent replicates. The Venn diagrams show the number of lipids that decreased (left), or increased (right), in each cell line with the degree of overlap.

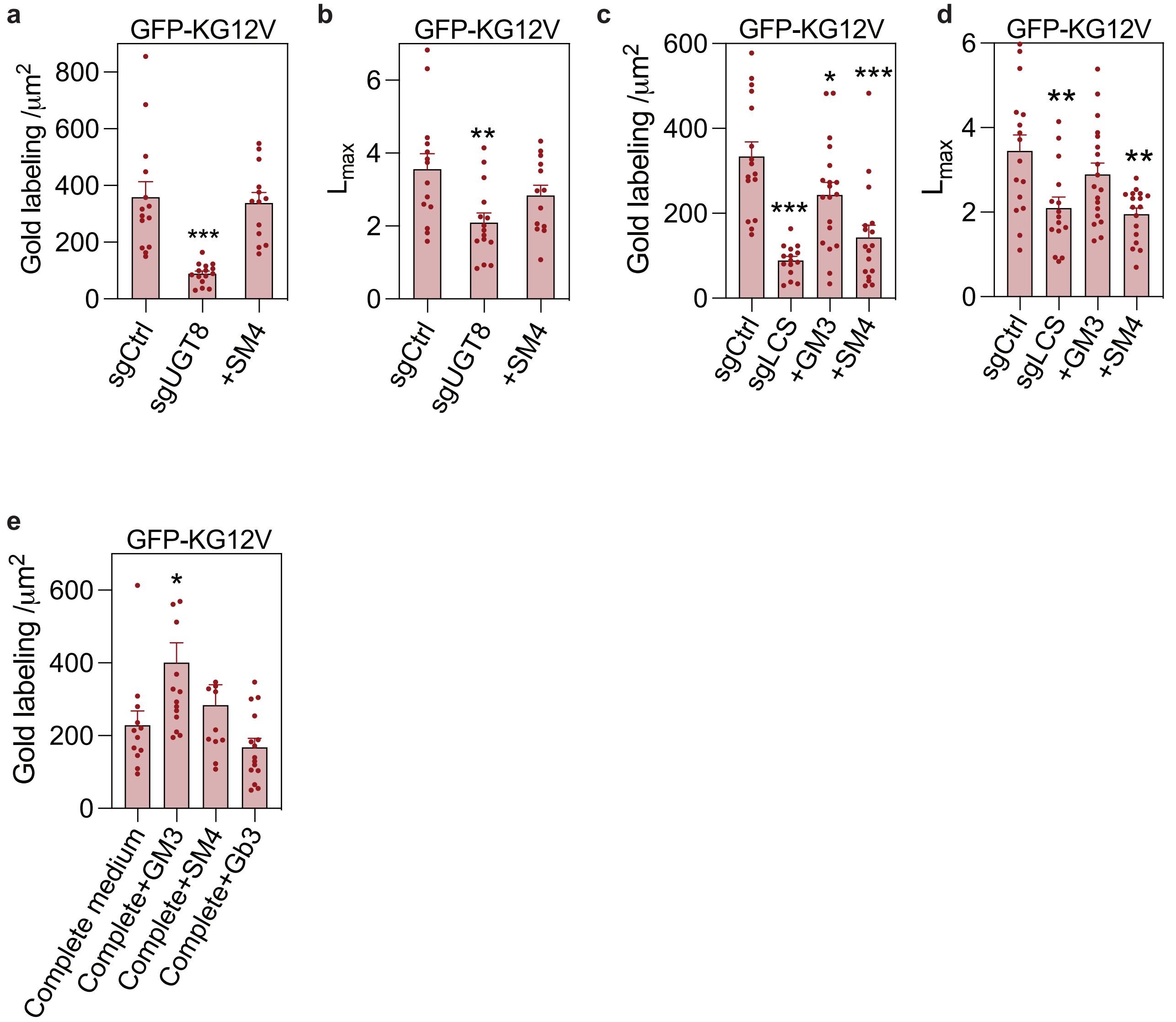
a
GFP-KG12V
Gold labeling /μm2
800
600
400
200
0
***
sgCtrl
sgUGT8
+SM4
b
GFP-KG12V
Lmax
6
4
2
0
**
sgCtrl
sgUGT8
+SM4
c
GFP-KG12V
Gold labeling /μm2
600
400
200
0
***
*
***
sgCtrl
sgLCS
+GM3
+SM4
d
GFP-KG12V
Lmax
6
4
2
0
**
**
sgCtrl
sgLCS
+GM3
+SM4
e
GFP-KG12V
Gold labeling /μm2
600
400
200
0
*
Complete medium
Complete+GM3
Complete+SM4
Complete+Gb3

Figure S5. Specific GSLs are required for KRAS PM localization

(**a-d**) Caco-2 cells stably expressing GFP-KRASG12V and deleted for UGT8 (sgUGT8) (=the first enzyme in SM4 biosynthesis) (n=14 PM sheets) (**a**, **b**), or LCS (sgLCS) (n=15) (**c**, **d**), and controls (sgCtrl) (n=16), were incubated with no GSL, or exogenous GM3 (n=19), or SM4 (n=17), for 1h as indicated. (**e**) GFP immunogold labeling density of PM sheets prepared from MDCK cells expressing GFP-KRASG12V cultured in normal glucose-replete medium and incubated with exogenous GM3, SM4 or Gb3 for 1hr. For (**a-e**), PM sheets prepared from the cells were immunogold labeled and imaged by EM. KRASG12V PM binding was quantified as mean gold labeling density (± SEM), and nanoclustering quantified using *Lmax* (± SEM). Significance differences between mean *L(r)-r* functions for GSL-addback and control cells were evaluated using bootstrap tests ($n \geq 15$, *$p < 0.05$), and differences in gold labeling density evaluated in t-tests (*$p<0.05$, **$p<0.01$).

Figure S6

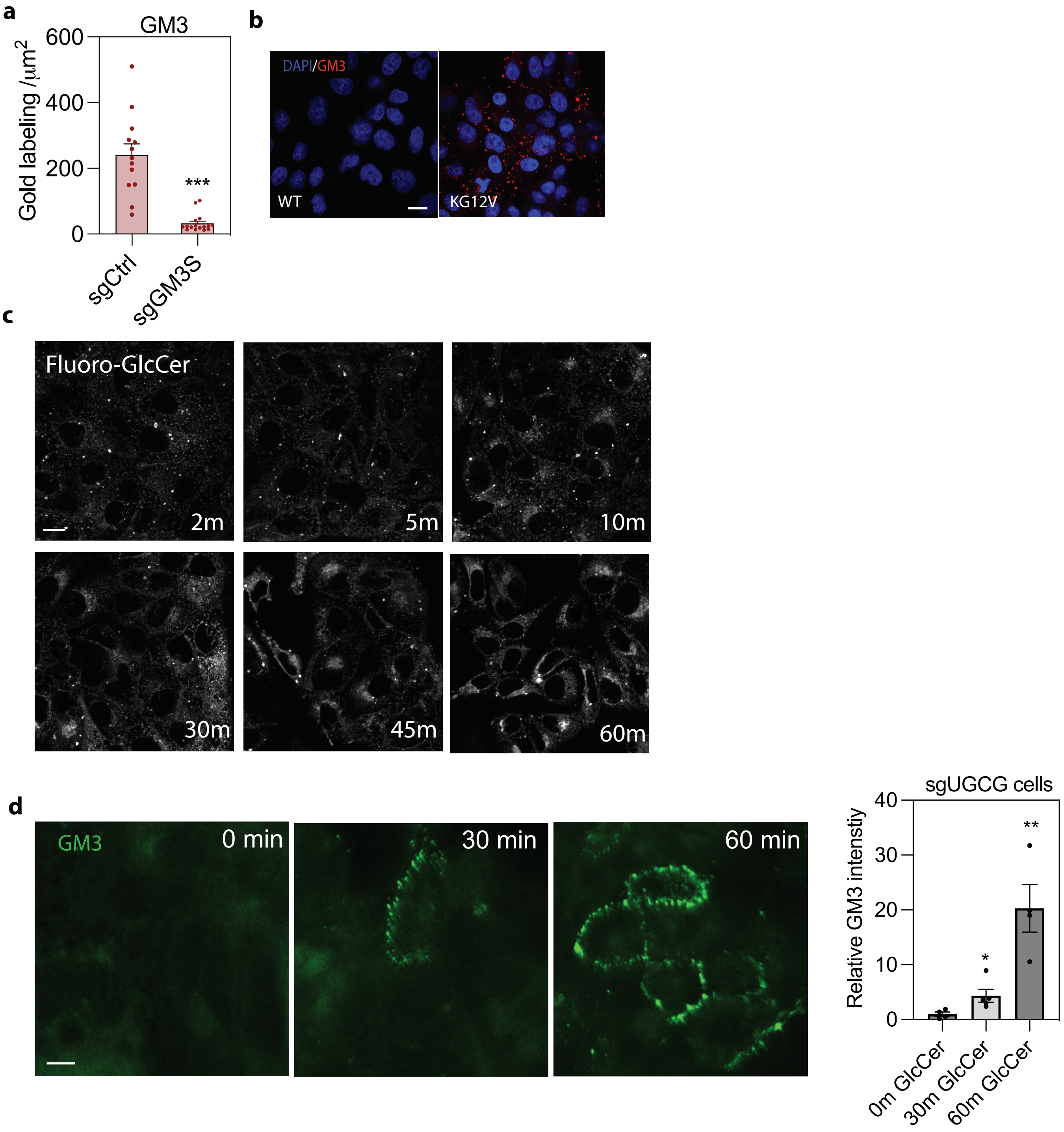
a
GM3
Gold labeling /μm2
600
400
200
0
***
sgCtrl
sgGM3S
b
DAPI/GM3
WT
KG12V
c
Fluoro-GlcCer
2m
5m
10m
30m
45m
60m
d
GM3
0 min
30 min
60 min
sgUGCG cells
Relative GM3 intenstiy
40
30
20
10
0
*
**
0m GlcCer
30m GlcCer
60m GlcCer

Figure S6. Validation of the anti-GM3 antibody and the addback assay

(**a**) PM sheets of control (sgCtrl) (n = 13) and GM3 synthase knockout (sgGM3S) (n = 16) Caco-2 cells were immunolabeled with anti-GM3 conjugated directly to 4.5 nm-gold particles. PM GM3 levels were quantified as gold labeling density. Data are shown as mean ± SEM ($n \geq 12$). Differences were evaluated using t-tests (**$p<0.01$). (**b**) Indirect immunohistochemical fluorescent staining of GM3 in WT and KRASG12V-expressing MDCK cells, using the same antibody as in A, and counterstained with DAPI. Compare with Figure 4H. Scale bar: 20μm. (**c**-**d**) We extensively validated lipid addback assays by several methods. First, our previous study using cell-expressed lipid probes showed that 1-hr incubation with exogenous PtdSer or PIP_2 was sufficient to reconstitute the inner PM with both species [8]. Secondly, (**c**) using Fluoro-GlcCer, we observed a rapid intake of GlcCer by MDCK cells in a time dependent manner, with clear accumulation in the endomembrane network at 30 mins post-incubation that stabilized at 45 mins. Scale bar: 20μm. Thirdly, (**d**) immunostaining analysis using an anti-GM3 antibody shows re-population of surface GM3 after 30min (n = 5 confocal images) and 1h GlcCer addback (n = 4) in UGCG knockout Caco-2 cells. Scale bar: 20μm. Differences in GM3 intensity were evaluated using two-tailed Student's t-tests (*$p<0.05$, **$p<0.01$). Representative pictures and quantification of immunostaining of GM3 in KRASG12V expressing Caco-2 cells depleted of UGCG and incubated with GlcCer for 0, 30 or 60 mins (±SEM, $n \geq 4$, *$p<0.05$).

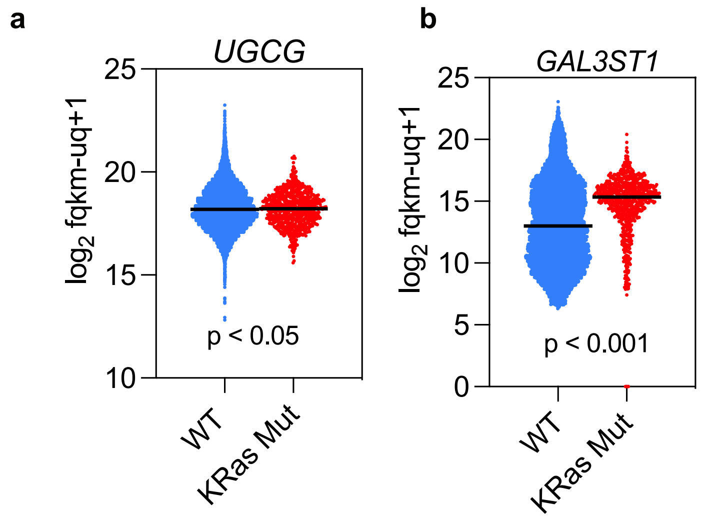

Figure S7.GSL synthetic enzymes in human cancer

Expression of (a) UGCG and (b) GAL3ST1 in KRAS WT (n = 11038) and mutant (n = 730) cancers. Welch's t-test was used to evaluate significant differences. RNA-seq data were acquired from GDC Pan-cancer database using the Xena browser.

Figure S8

a

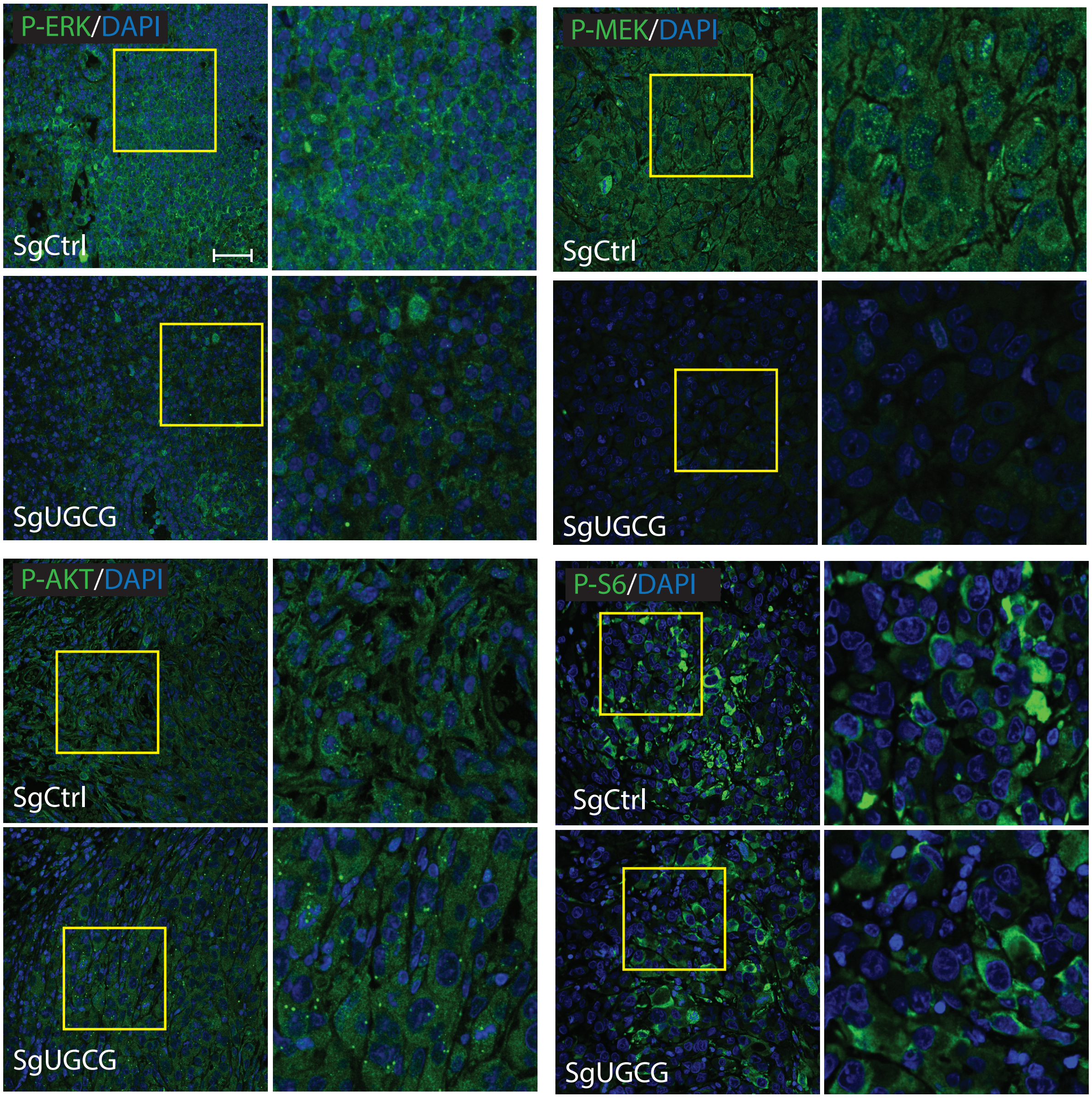
P-ERK/DAPI
SgCtrl
SgUGCG
P-MEK/DAPI
SgCtrl
SgUGCG
P-AKT/DAPI
SgCtrl
SgUGCG
P-S6/DAPI
SgCtrl
SgUGCG

b

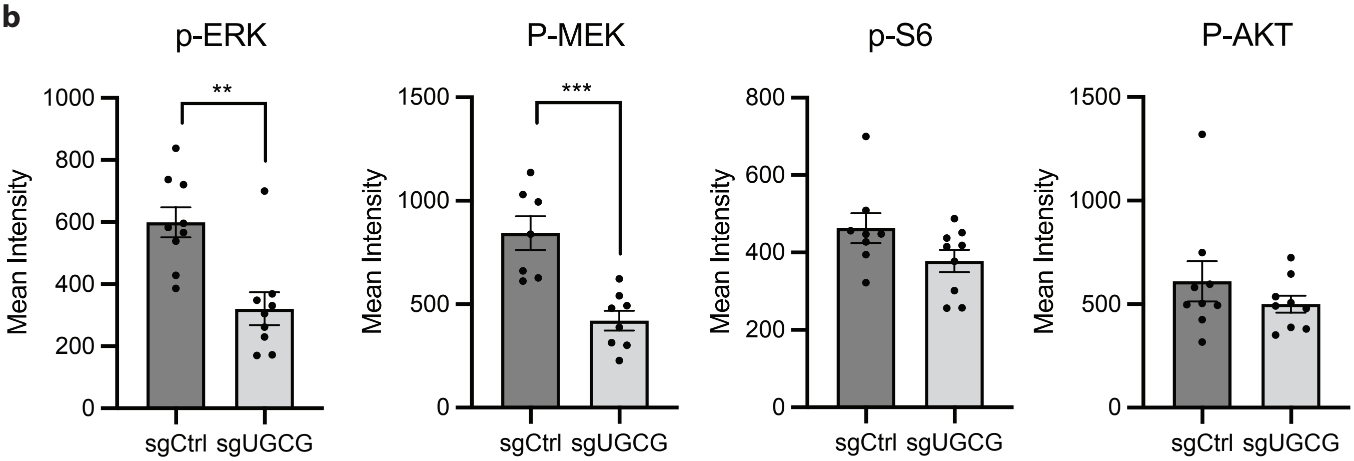
p-ERK
P-MEK
p-S6
P-AKT
Mean Intensity
**
***
sgCtrl
sgUGCG
0
200
400
600
800
1000
500
1000
1500

Figure S8. UGCG deletion impairs MAPK signaling in MIA PaCa-2 xenografts

(**a**, **b**) Tumor sections of MIA PaCa-2 cells depleted of UGCG (sgUGCG) (n=3) or expressing control vector (sgCtrl) (n=3) were stained with DAPI and indicated antibodies. (**a**) Representative images for each antibody are shown. Rectangular regions enclosed by yellow boxes are highlighted in the right panels at high magnification. Scale bar: 50mm. (**b**) Quantification of staining intensity for each protein was calculated using multiple images for each tumor (±SEM, *$p<0.05$, n=9 images analyzed for p-ERK, n=7 for p-MEK, n=9 for p-AKT, n=8 for p-S6K).

a

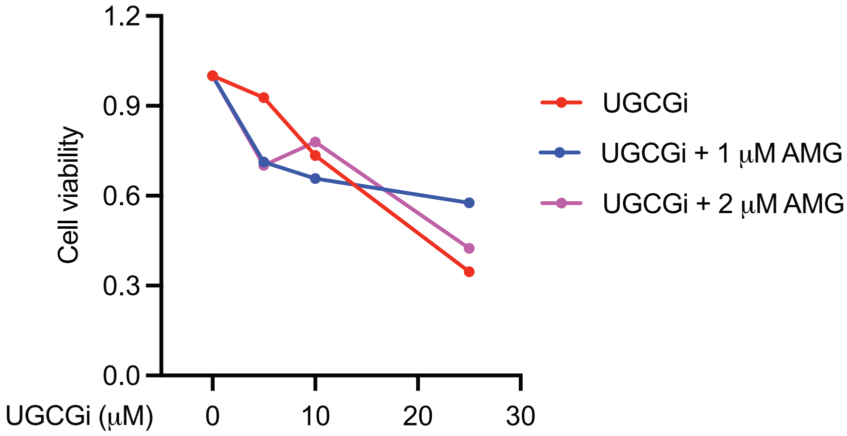

b

AMG510 (μM)	UGCGi (μM)	Cell viability (%)	CI
1	5	0.71	1.06
1	10	0.65	1.06
1	25	0.57	1.85
2	5	0.72	1.89
2	10	0.77	3.81
2	25	0.42	1.25

Figure S9. Lack of synergy between a UGCG inhibitor and a direct KRAS G12C inhibitor

$5x10^3$ MIA PaCa-2 cells were seeded in each well of a 96-well plate on day 1. The next day the cells were treated with a KRASG12C or UGCG inhibitor, or two inhibitors combined at different concentrations. (a) After 72hr of incubation cell viability was analyzed by the Cyquant assay. (b) Combination index (CI) was calculated based on the effects of each single agent on cell viability and combined effects of two inhibitors. By this method a CI <1 indicates synergism, a CI > 1 indicates antagonism and CI of 1 indicates an additive effect.
