# Supplementary material for: Glycolysis regulates KRAS plasma membrane localization and function through defined glycosphingolipids
Source: Nat Commun. 2023 Jan 28;14:465. doi: 10.1038/s41467-023-36128-5 (PMC9884228; doi:10.1038/s41467-023-36128-5)
Supplement: Supplementary file 3 — Reporting Summary [file 41467_2023_36128_MOESM3_ESM.pdf]

nature portfolio

Corresponding author(s): John F Hancock

Last updated by author(s): Jan 12, 2022

Reporting Summary

Nature Portfolio wishes to improve the reproducibility of the work that we publish. This form provides structure for consistency and transparency in reporting. For further information on Nature Portfolio policies, see our Editorial Policies and the Editorial Policy Checklist.

Statistics

For all statistical analyses, confirm that the following items are present in the figure legend, table legend, main text, or Methods section.

n/a	Confirmed	
☐	☒	The exact sample size (*n*) for each experimental group/condition, given as a discrete number and unit of measurement
☐	☒	A statement on whether measurements were taken from distinct samples or whether the same sample was measured repeatedly
☐	☒	The statistical test(s) used AND whether they are one- or two-sided<br>*Only common tests should be described solely by name; describe more complex techniques in the Methods section.*
☒	☐	A description of all covariates tested
☒	☐	A description of any assumptions or corrections, such as tests of normality and adjustment for multiple comparisons
☐	☒	A full description of the statistical parameters including central tendency (e.g. means) or other basic estimates (e.g. regression coefficient) AND variation (e.g. standard deviation) or associated estimates of uncertainty (e.g. confidence intervals)
☐	☒	For null hypothesis testing, the test statistic (e.g. *F*, *t*, *r*) with confidence intervals, effect sizes, degrees of freedom and *P* value noted<br>*Give P values as exact values whenever suitable.*
☒	☐	For Bayesian analysis, information on the choice of priors and Markov chain Monte Carlo settings
☒	☐	For hierarchical and complex designs, identification of the appropriate level for tests and full reporting of outcomes
☒	☐	Estimates of effect sizes (e.g. Cohen's *d*, Pearson's *r*), indicating how they were calculated

Our web collection on statistics for biologists contains articles on many of the points above.

Software and code

Policy information about availability of computer code

Data collection	Commercially available software have been used in the study and described in the methods section of the manuscript. ImageJ, Graphpad Prism 9.0 and Cell Counter.
Data analysis	Graphpad 9.0, Image J 2.0 have been used for data analysis. Codes used in this study including K_Function_UNIVARIATE, K_Function_BIVARIATE and BIV_LOOKUP_TABLE, have been deposited by the authors to Figshare (https://figshare.com/articles/software/Liu_et_al_2022_Nat_Co_codes/21685151).

For manuscripts utilizing custom algorithms or software that are central to the research but not yet described in published literature, software must be made available to editors and reviewers. We strongly encourage code deposition in a community repository (e.g. GitHub). See the Nature Portfolio guidelines for submitting code & software for further information.

Data

Policy information about availability of data

All manuscripts must include a data availability statement. This statement should provide the following information, where applicable:

- Accession codes, unique identifiers, or web links for publicly available datasets
- A description of any restrictions on data availability
- For clinical datasets or third party data, please ensure that the statement adheres to our policy

: The lipidomics data generated in this study have been deposited to Figshare (https://figshare.com/articles/dataset/Liu_et_al_lipidomics_xlsx/21678833). The

source data underlying all main figures and supplementary figures are provided as a source data file. Specific p-values are also included in the source data file. All data are available in the main article, Supplementary Information and source data.

Human research participants

Policy information about studies involving human research participants and Sex and Gender in Research.

Reporting on sex and gender	*Use the terms sex (biological attribute) and gender (shaped by social and cultural circumstances) carefully in order to avoid confusing both terms. Indicate if findings apply to only one sex or gender; describe whether sex and gender were considered in study design whether sex and/or gender was determined based on self-reporting or assigned and methods used. Provide in the source data disaggregated sex and gender data where this information has been collected, and consent has been obtained for sharing of individual-level data; provide overall numbers in this Reporting Summary. Please state if this information has not been collected. Report sex- and gender-based analyses where performed, justify reasons for lack of sex- and gender-based analysis.*
Population characteristics	*Describe the covariate-relevant population characteristics of the human research participants (e.g. age, genotypic information, past and current diagnosis and treatment categories). If you filled out the behavioural & social sciences study design questions and have nothing to add here, write "See above."*
Recruitment	*Describe how participants were recruited. Outline any potential self-selection bias or other biases that may be present and how these are likely to impact results.*
Ethics oversight	*Identify the organization(s) that approved the study protocol.*

Note that full information on the approval of the study protocol must also be provided in the manuscript.

Field-specific reporting

Please select the one below that is the best fit for your research. If you are not sure, read the appropriate sections before making your selection.

☐ Life sciences ☐ Behavioural & social sciences ☐ Ecological, evolutionary & environmental sciences

For a reference copy of the document with all sections, see nature.com/documents/nr-reporting-summary-flat.pdf

Life sciences study design

All studies must disclose on these points even when the disclosure is negative.

Sample size	For mouse pancreatic cancer experiments, ten mice of each condition were used. For all in vivo experiments, the sample size was chosen to generate a 90% power to detect a 20% difference with an estimated variability of 20% using a two-sided t-test at a 5% significance level.
Data exclusions	For in vivo experiments, no data were excluded; in rare cases, a single value of multiple replicates was excluded when known technical or measurement errors were present.
Replication	Cell viability assays have been repeated in triplicate. For soft agar assay more than three replicates for each group were included in the study to increase the rigor. For electron microscopy experiments, more than 12 images of plasma membrane ripoffs for each condition were analyzed.
Randomization	For mouse cancer models, mice were randomly separated into two groups and were implanted with control or knockout KPC cells. For other assays, the experimental groups were equally distributed therefore no randomization process was performed.
Blinding	For pancreatic caner models, researchers typically do not know the treatment of each mouse when measuring tumor size as the mice were randomly numbered.

Behavioural & social sciences study design

All studies must disclose on these points even when the disclosure is negative.

Study description	*Briefly describe the study type including whether data are quantitative, qualitative, or mixed-methods (e.g. qualitative cross-sectional, quantitative experimental, mixed-methods case study).*
Research sample	*State the research sample (e.g. Harvard university undergraduates, villagers in rural India) and provide relevant demographic information (e.g. age, sex) and indicate whether the sample is representative. Provide a rationale for the study sample chosen. For studies involving existing datasets, please describe the dataset and source.*
Sampling strategy	*Describe the sampling procedure (e.g. random, snowball, stratified, convenience). Describe the statistical methods that were used to*

Sampling strategy	*predetermine sample size OR if no sample-size calculation was performed, describe how sample sizes were chosen and provide a rationale for why these sample sizes are sufficient. For qualitative data, please indicate whether data saturation was considered, and what criteria were used to decide that no further sampling was needed.*
Data collection	*Provide details about the data collection procedure, including the instruments or devices used to record the data (e.g. pen and paper, computer, eye tracker, video or audio equipment) whether anyone was present besides the participant(s) and the researcher, and whether the researcher was blind to experimental condition and/or the study hypothesis during data collection.*
Timing	*Indicate the start and stop dates of data collection. If there is a gap between collection periods, state the dates for each sample cohort.*
Data exclusions	*If no data were excluded from the analyses, state so OR if data were excluded, provide the exact number of exclusions and the rationale behind them, indicating whether exclusion criteria were pre-established.*
Non-participation	*State how many participants dropped out/declined participation and the reason(s) given OR provide response rate OR state that no participants dropped out/declined participation.*
Randomization	*If participants were not allocated into experimental groups, state so OR describe how participants were allocated to groups, and if allocation was not random, describe how covariates were controlled.*

Ecological, evolutionary & environmental sciences study design

All studies must disclose on these points even when the disclosure is negative.

Study description	*Briefly describe the study. For quantitative data include treatment factors and interactions, design structure (e.g. factorial, nested, hierarchical), nature and number of experimental units and replicates.*
Research sample	*Describe the research sample (e.g. a group of tagged Passer domesticus, all Stenocereus thurberi within Organ Pipe Cactus National Monument), and provide a rationale for the sample choice. When relevant, describe the organism taxa, source, sex, age range and any manipulations. State what population the sample is meant to represent when applicable. For studies involving existing datasets, describe the data and its source.*
Sampling strategy	*Note the sampling procedure. Describe the statistical methods that were used to predetermine sample size OR if no sample-size calculation was performed, describe how sample sizes were chosen and provide a rationale for why these sample sizes are sufficient.*
Data collection	*Describe the data collection procedure, including who recorded the data and how.*
Timing and spatial scale	*Indicate the start and stop dates of data collection, noting the frequency and periodicity of sampling and providing a rationale for these choices. If there is a gap between collection periods, state the dates for each sample cohort. Specify the spatial scale from which the data are taken*
Data exclusions	*If no data were excluded from the analyses, state so OR if data were excluded, describe the exclusions and the rationale behind them, indicating whether exclusion criteria were pre-established.*
Reproducibility	*Describe the measures taken to verify the reproducibility of experimental findings. For each experiment, note whether any attempts to repeat the experiment failed OR state that all attempts to repeat the experiment were successful.*
Randomization	*Describe how samples/organisms/participants were allocated into groups. If allocation was not random, describe how covariates were controlled. If this is not relevant to your study, explain why.*
Blinding	*Describe the extent of blinding used during data acquisition and analysis. If blinding was not possible, describe why OR explain why blinding was not relevant to your study.*

Did the study involve field work? ☐ Yes ☐ No

Field work, collection and transport

Field conditions	*Describe the study conditions for field work, providing relevant parameters (e.g. temperature, rainfall).*
Location	*State the location of the sampling or experiment, providing relevant parameters (e.g. latitude and longitude, elevation, water depth).*
Access & import/export	*Describe the efforts you have made to access habitats and to collect and import/export your samples in a responsible manner and in compliance with local, national and international laws, noting any permits that were obtained (give the name of the issuing authority, the date of issue, and any identifying information).*
Disturbance	*Describe any disturbance caused by the study and how it was minimized.*

Reporting for specific materials, systems and methods

We require information from authors about some types of materials, experimental systems and methods used in many studies. Here, indicate whether each material, system or method listed is relevant to your study. If you are not sure if a list item applies to your research, read the appropriate section before selecting a response.

Materials & experimental systems

n/a	Involved in the study
☐	☒ Antibodies
☐	☒ Eukaryotic cell lines
☒	☐ Palaeontology and archaeology
☐	☒ Animals and other organisms
☒	☐ Clinical data
☒	☐ Dual use research of concern

Methods

n/a	Involved in the study
☒	☐ ChIP-seq
☒	☐ Flow cytometry
☒	☐ MRI-based neuroimaging

Antibodies

Antibodies used

The anti-p-ERK (1:1000), anti-p-MEK (1:1000), anti-total-ERK (1:1000), anti-total MEK (1:1000), anti-actin antibodies were (1:4000) purchased from CELL Signaling Technology. GM3, Anti-GFP and anti-RFP antibodies used for immunogold labeling were made in house, and anti-GM3 antibodies used for immunogold and immunofluorescent labeling were purchased from Amsbio LLC (GMR6).

Validation

All primary antibodies were validated by a positive and negative control samples. For gold conjugated GM3 antibodies that were used in EM analyses, plasma membrane sheets of human Caco-2 cells depleted of GM3 synthase (st3gal5) were used as a positive control to validate the absence of GM3 in knockout cells. To validate p-ERK and p-MEK antibodies Caco-2 were starved with serum for 1h, and probed with respective antibodies to confirm the reduction of the bands. GFP and RFP antibodies were verified in multiple previous publications (eg. PMID: 12684529).

Eukaryotic cell lines

Policy information about cell lines and Sex and Gender in Research

Cell line source(s)

Mardin-Darby canine kidney (MDCK) cells, Gift of Dr. Robert G. Parton (University of Queensland, Australia); KPC cells, gift of Dr. Jennifer Bailey, McGovern Medical School, Houston; Caco-2, MiaPaCa-2 and PANC-1 cells were purchased from American Type Culture Collection; BxPC3 and MOH were provided by Dr. Craig Logsdon at MD Anderson Cancer, Houston, TX.

Authentication

All cell lines were validated by STR analysis by the vendors or donors.

Mycoplasma contamination

Each cell line used in this study was tested and free of mycoplasma.

Commonly misidentified lines
(See ICLAC register)

No misidentified cell lines have been used in this study.

Animals and other research organisms

Policy information about studies involving animals; ARRIVE guidelines recommended for reporting animal research, and Sex and Gender in Research

Laboratory animals

4-8 week old female nu/nu (#007850-Outbred athymic nude) and male C57BL/6 (#000664-C57BL/6J inbred) mice were purchased from The Jackson Laboratory (Bar Harbor, ME). The mice were housed in a facility at room temperature with 12-hour dark and light cycle. All animals were euthanized by introduction of 100% carbon dioxide to the home cages, followed by cervical dislocation.

Wild animals

The study does not involve wild animals.

Reporting on sex

Sex of the mice could be a relevant biological variable for mouse pancreatic cancer models. We used female nu/nu (#007850-Outbred athymic nude) and male C57BL/6 (#000664-C57BL/6J inbred) mice for the study.

Field-collected samples

The study does not involve field-collected samples.

Ethics oversight

All animal studies were performed under an Institutional Animal Care and Use Committee (IACUC) approved animal protocol, in accordance with the National Institutes of Health Guide for the Care and Use of Laboratory Animals.

Note that full information on the approval of the study protocol must also be provided in the manuscript.
